# Tryptophan status in autism spectrum disorder and the influence of supplementation on its level

**DOI:** 10.1007/s11011-017-0045-x

**Published:** 2017-06-12

**Authors:** Joanna Kałużna-Czaplińska, Jagoda Jóźwik-Pruska, Salvatore Chirumbolo, Geir Bjørklund

**Affiliations:** 1 Department of Chemistry, Institute of General and Ecological Chemistry, Lodz University of Technology, Zeromskiego116, 90-924 Lodz, Poland; 2 Department of Neurological and Movement Sciences, University of Verona, Verona, Italy; 3 Council for Nutritional and Environmental Medicine, Mo i Rana, Norway

**Keywords:** Autism, Tryptophan, Amino acid, Supplementation, Urine, Chromatography

## Abstract

Recent reports show that the worldwide incidence of autism spectrum disorder (ASD) is dramatically increasing, although ASD etiology and pathogenesis are still far to be fully elucidated. Some dietary-derived essential compounds, such as the amino acid tryptophan, appear to be impaired in patients with ASD. Tryptophan (Trp) plays a significant role in the human organism and serves as a precursor for a wide range of bioactive compounds, including major neurotransmitters. Research indicates that tryptophan might be deficient in subjects with ASD. Deficiency in the tryptophan level can be retrieved by investigating Trp levels or its major metabolite kynurenine in urines. The purpose of the present study is to quantify tryptophan content in urine samples (*n* = 236) of ASD patients, who underwent a supplemented dietary panel with B vitamins and magnesium, compared to controls (without this diet regimen). The samples were analyzed with gas chromatography-mass spectrometry. Additionally, the correlation between body mass index (BMI) and the level of this amino acid in urine was accomplished. Basic parameters of urine samples were also evaluated. Statistical evaluations in the concentration of tryptophan in ASD patients with different severity of symptoms were reported. A significant difference in tryptophan levels in all groups was observed. Supplementation with B vitamins and magnesium has an influence on the Trp concentration. Furthermore, no correlation between BMI and tryptophan levels was found. These results assess that the Trp level in ASD subjects is critical and that intake of B vitamins and magnesium with diet might influence its metabolic homeostasis.

## Introduction

L-tryptophan is an essential amino acid, which plays a significant role even in neurodegeneration if its catabolism is functionally impaired (Morris et al. 2016a, b). Renal excretion of tryptophan and its pharmacokinetics in humans has been investigated in past reports and should depend on the temporal occurrence of kynurenine and 3-hydroxykynurenine in plasma and urine, associated with the effect of nicotinamide (Møller 1981). The increase in the indolamine-2,3-dioxygenase (IDO), the tryptophan-metabolizing enzyme, has been associated with immunological and inflammatory disorders, at least in animal models (Wirthgen et al. 2016). Actually, tryptophan levels in urine can help researchers to highlight the relationship between this amino acid and its relationship with catabolites such as kynurenine with neurological and cognitive disorders associated with immune-related pathology (Gabbay et al. 2010; Keegan et al. 2016). The relationship between tryptophan and neurological disorders may be explained by the evidence that this amino acid is a known precursor of fundamental neuromodulators, and can even explain the recent evidence about its therapeutical use in neuroscience, despite the existence of controversial results (Wang et al. 2016; Nagashima et al. 2017; Silva et al. 2017). Precursors of L-tryptophan can be found in eggs, meat, cereal, milk, bananas, fish, seafood and plums, though the amino acid is also synthesized by gut microflora, which should contribute to altered tryptophan metabolism, yielding increased levels of indolyl 3-acetic acid and indolyl lactate (Richard et al. 2009; Gevi et al. 2016). Tryptophan plays a role in the serotonin and kynurenine pathway, protein synthesis, and bacterial degradation, as current literature reported evidence about the role of tryptophan metabolites, especially serotonin, in the pathophysiology of the gut, through the many mechanisms underlying these connections are still far to be elucidated (Peters 1991; Keszthelyi et al. 2009; Gevi et al. 2016).

It has been estimated that only 1–2% of tryptophan is metabolized into the neurotransmitter serotonin, which is also known as a precursor of melatonin. The great amount of the body tryptophan assessed by its bioavailability enters the kynurenine pathway and leads to the production of kynurenic acid, kynuramines, picolinic acid, NAD, and ATP (Fernstrom 2016). Regulation of plasma tryptophan, maintenance of nicotinic acid level, clearance of excess tryptophan, regulation of CNS function and participation in the immune system response are some of the most important biological functions of this pathway (Peters 1991; O'Mahony et al. 2015; Jenkins et al. 2016). The amount of tryptophan, which is secreted in unchanged form from the body, is estimated as low as 0.5% (Keszthelyi et al. 2009).

Impaired functioning of the central nervous system, sleep disturbances, immune dysregulation, metabolic disorders, anxiety attacks, impaired communication and social interaction are just some of the symptoms of autism spectrum disorder (ASD) (Volkmar et al. 2005; Kanne et al. 2009; van Steensel et al. 2011; Vriend et al. 2011; Frye and Rossignol 2012). In the more recent years, research has shown a dramatic increase in the incidence of ASD. What’s more, the disorder was officially recognized as one of the most serious health problems of the world next to AIDS, cancer, and diabetes (Centers for Disease Control and Prevention, 2017).

At least one of 68 children in the US has ASD (Zablotsky et al. 2015; Christensen et al. 2016). Although the direct cause of the increase in the prevalence of ASD is unknown, it can partly be explained due to increased awareness and improvement in diagnosis. ASD is more often diagnosed in boys (1 to 42) than girls (1 to 189) (Autism and Developmental Disabilities Monitoring Network Surveillance Year 2010 Principal Investigators, 2014).

The etiology of ASD is still unclear. Most of the scientific investigations are focused on the genetic aspect of this disorder (Egawa et al. 2013). Less emphasis is placed on defining the importance of the many different environmental factors. It should be highlighted that heredity does not explain all of the reported cases and the drastic increase in the number of incidence in recent decades. Also, the twin studies have shown that environmental factors account for 55% risk of developing ASD, and genetic factors can only explain 37% of the cases (Shaw et al. 2014).

The relationship between tryptophan bio-organic levels in humans and ASD as coming in the spotlight in the very recent years (Schwartz 2014; Zheng et al. 2017). Impairments in the brain-gut axis may involve tryptophan metabolism and induce disorders in immunity and neurological function or moods (Morris et al. 2016b).

Furthermore, serotonergic abnormalities were reported in ASD (Anderson 1994; Politte et al. 2014). Research indicates that the reduced levels of serotonin are correlated with the occurrence of impulsive and aggressive behavior, fatigue, depression episodes, insomnia and increased sensitivity to pain (Keszthelyi et al. 2009). There is evidence that pharmacological treatment is aiming the reduction of the serotonergic neurotransmission results in worsening of autistic symptoms (McDougle et al. 1996; Politte et al. 2014; Accordino et al. 2016). On the other hand, the introduction of serotonin reuptake inhibitors may lead to the alleviation of compulsive symptoms, motor stereotypies, and facilitate the social functioning of adult individuals with ASD, though controversial opinions yet exist about possible adverse effects of this therapy (Chugani et al. 1999; Kaplan et al. 2016; Kobayashi et al. 2016; Zhang et al. 2017).

Children with ASD are known to have disturbed production of melatonin, which is formed on serotonin pathway from tryptophan. Melatonin is responsible for the regulation of circadian rhythms (Rossignol and Frye 2011).

Moreover, gastrointestinal disorders in individuals with ASD have been described already in the 70s of the last century. The way in which gastrointestinal disturbances are correlated with the presence and severity of autistic symptoms is still unclear, although interesting attempts were approached (Goodwin et al. 1971; Johnston 2000; Frye et al. 2015; Krajmalnik-Brown et al. 2015; Kang et al. 2017).

The role of tryptophan (Trp) in ASD came in the spotlight when researchers asked how much diet micronutrients or their reduced availability may affect the exacerbation or progress of this neurological and mood disorder (Boccuto et al. 2013; Gevi et al. 2016; Essa et al. 2013). In the present study, the results of a research investigation focused on the tryptophan retention status in subjects with ASD are presented. Additionally, the purpose of the present study was to describe the correlation between received supplementation (B vitamins and magnesium) and the level of the excreted (excess) tryptophan in ASD patients. The correlation between the concentration and BMI was also examined.

## Materials and methods

### Patients and sample collection

The study was restricted to children with a diagnosis of ASD in compliance with the criteria detailed in the Diagnostic and Statistical Manual of Mental Disorders (American Psychiatric Association, 2000). The population was divided into groups due to the type of disorder associated with the severity of ASD symptoms: autistic disorder patients, Asperger syndrome patients, and autistic disorder patients with psychomotor retardation. All overnight urine samples were collected from 236 ASD children (3–16 years, sex equally distributed), who underwent rehabilitation at the Clinic of Developmental of Dislocation Navicula-Centre in Lodz. Of these children had 188 autistic disorder and 48 had Asperger syndrome. Of the children with autistic disorder had 37 psychomotor retardation. The cohort stratification is described in Table 1. All ASD children were assessed and diagnosed by specialty clinicians with expertise in the diagnosis and management of ASD children from the Navicula. The ASD children were not subjected to a gluten-free, casein-free or sugar-free diet. The study was approved by the Review Board of the Institute and performed in agreement with the Standards and Ethics in Biological Rhythm Research (Portaluppi et al. 2010). Urine was collected and stored at −20 °C until analysis.

**Table 1:** Stratification of the tested population

ASD children	children	*n = 236*
	boys	189
	girls	47
Autistic disorder	vitamins B and Mg	18
	omega-3 fatty acids	38
Asperger syndrome	vitamins B and Mg	23
	omega-3 fatty acids	17
Autistic disorder and psychomotor retardation	vitamins B and Mg	20
	omega-3 fatty acids	29

Additionally, basic parameters of each urine sample were evaluated, including specific gravity (SG), pH, leukocytes (LEU), nitrite (NIT), protein (PRO), glucose (GLU), ketone (KET), urobilinogen (UBG), bilirubin (BIL), erythrocytes (BLD). For this purpose, urine analyzer Urisys 1100 was used. Reference values are listed in Table 2.

**Table 2:** Reference values for urine samples (adapted from Simerville et al. 2005)

Parameter	SG	pH	LEU	NIT	PRO	GLU	KET	UBG	BIL	BLD
Reference value	1.016–1.022 kg/l	4.6–7.0	neg	neg	neg	norm	neg	norm	neg	neg

neg = absent; norm = within the normal range

A cohort of subjects with autistic disorder underwent supplementation with B vitamins and magnesium, while children with autistic disorder without supplementation were introduced in the study as their matched controls. A second cohort of patients with Asperger syndrome underwent the same experimental sub-grouping: a) subjects with supplementation of B vitamins and magnesium; b) subjects without supplementation (controls). The body mass index (BMI) was calculated for all patients (Table 3).

**Table 3:** Characterisation of the population based on body mass index (BMI) value

BMI ranges	% of population	Category
<18.5	87	Underweight
18.5–24.9	11	Normal (healthy weight)
25.0–29.9	2	Overweight
30.0–40.0	0	Obese
>40.0	0	Extremely obese

### Chemicals and reagents

Ethanol and chloroform were obtained from Sigma-Aldrich (Germany). Pyridine was purchased from Chempur (Poland), and ethylchloroformate was obtained from Fluka (Switzerland). As the internal standard (IS), the compound 4-chloro-L-phenylamine (Sigma-Aldrich, Germany) in chloroform was used. All chemicals were pure and of HPLC solvent grade.

### Sample preparation and analytical methods

For sample preparation, a simple modification of the method previously described was used (Kałużna-Czaplińska et al. 2010). Briefly, tryptophan was isolated from urine samples and derivatized before the chromatographic analysis. Applied method enables simultaneous extraction and derivatization of analytes.

Urine samples (0.5 ml) were transferred to centrifuge tubes, and 100 μl of IS (1.0 mg/ml) was added. Samples were extracted and derivatized with the addition of 600 μl of chloroform, 100 μl of pyridine and 60 μl of ethylchloroformate. Samples were vortex-mixed, and the organic phase (lower) was taken as an extract. Analyses of tryptophan were performed with the application of gas chromatography-mass spectrometry.

The results are expressed as ratios to the urinary creatinine concentration in μmol/mmol of creatinine. Creatinine was determined by the use of a high-performance liquid chromatography method reported elsewhere (Kuśmierek et al. 2006).

### GC-MS analysis

An aliquot of 1.0 μl of a prepared sample was injected splitless into an Agilent 6890 N Network GC system and 5973 Network Mass Selective equipped with a capillary column (J&W Ultra Inert HP-5 ms; Agilent Technology; 30 m × 0.25 mm internal diameter; film thickness, 0.25 m). The injector temperature was set at 290 °C. Helium was used as a carrier gas at a constant flow rate of 0.9 mL/min through the column. The column temperature was initially kept at 100 °C for 1 min and then increased to 180 °C at 20 °C min^−1^, and to 250 °C at 10 °C min^−1^ and further increased at 5 °C min^−1^ to 265 °C. The MS quadrupole temperature was set at 230 °C. Masses were acquired from m/z 50–400. MassHunter Workstation Software was used to identify and quantify tryptophan.

### Basic parameters evaluation

Urisys 1100 is a reflectance photometer, which reads and evaluates the urine test strips. Test strips were dipped in urine samples, and the excess of urine was wiped off. The strip was placed in the analyzer, and the measurement was performed.

### Statistical analysis

Data were statistically evaluated using statistical analysis package (StatSoft, Poland STATISTICA, version 9.0.). The Shapiro-Wilk test was used to check for normal distribution of the results. When non-parametric, the Mann–Whitney *U* test was used to determine differences between values of tryptophan in both groups. The level of statistical significance was defined as *p* < 0.05.

## Results

**Fig. 1:**
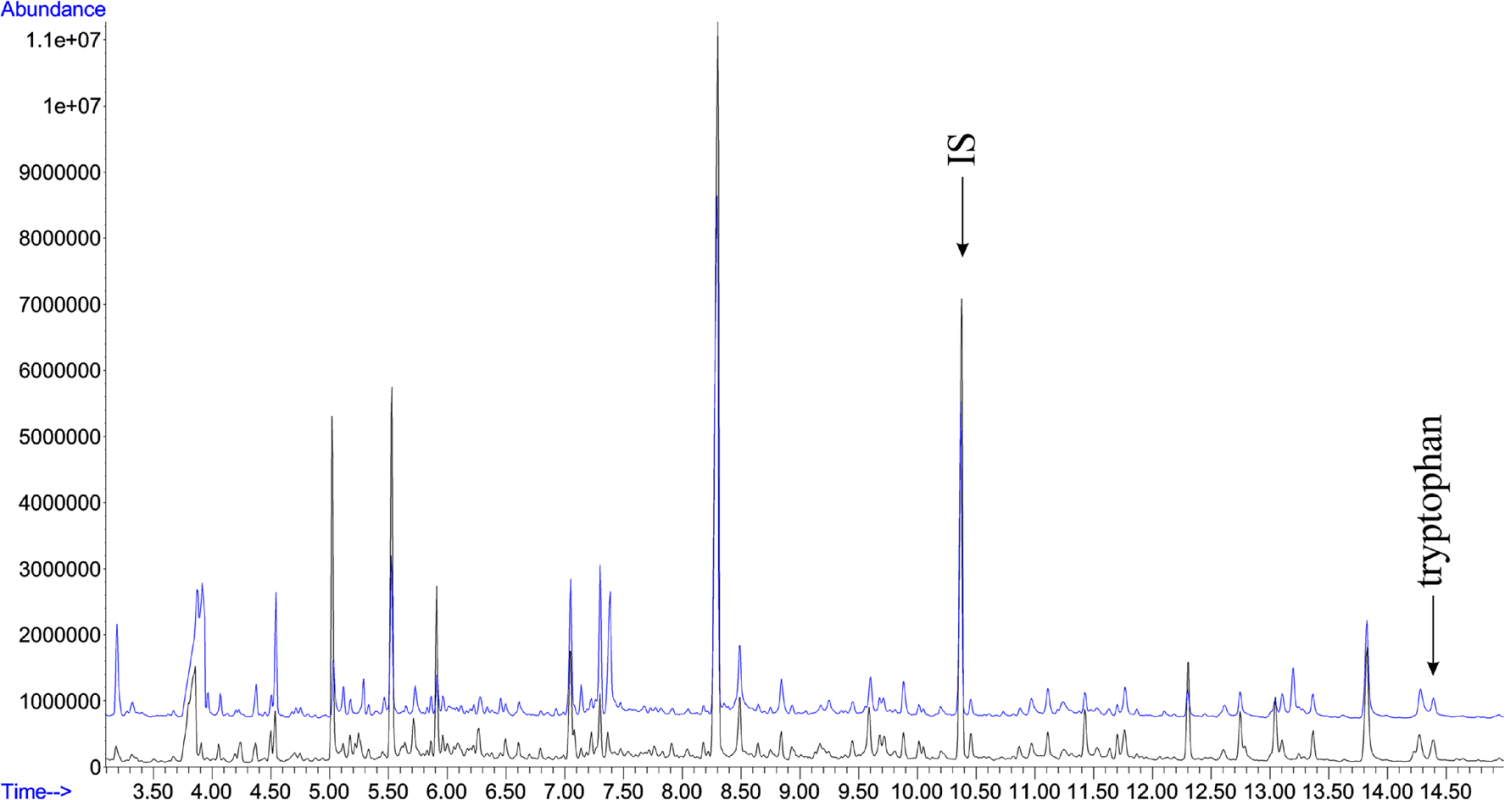
Overlap of two typical TIC amino acid profiles from the total assessed in the study

In the present study, a simple, precise, sensitive, and non-invasive gas chromatography/mass spectrometry method for the evaluation of tryptophan in urine samples was applied. To assess the stability of the analytical system, a quality control sample was performed during GC–MS analyses. Figure 1 presents an example of the overlap of two typical TIC amino acids profiles of injected autistic urine samples in the same aliquot. The data showed stable retention time with no drift of the peaks, which reflects the stability of GC–MS analysis and reliability of the metabolomic data.

According to the data published on Human Metabolome Database, the reference values for tryptophan concentrations ranges from 2.04 to 27.02 μmol/mmol creatinine for the entire considered age bracket (Wishart et al. 2013).

**Fig. 2:**
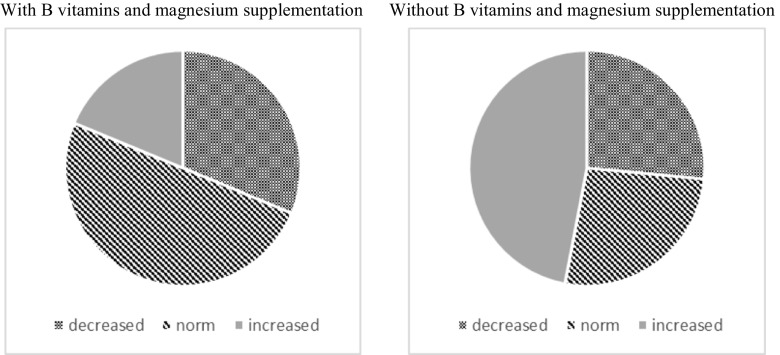
Comparison of obtained results in both study groups. In these pie charts it is evident how about one half of the investigated patients decreased levels of tryptophan excretion following a vitamin B and Mg^++^ supplementation diet (*left*), while the prevention of this diet intake and the reduction of vitamin B and magnesium assumption with nutrients, caused an increase in Trp urine content (*right*)

**Fig. 3:**
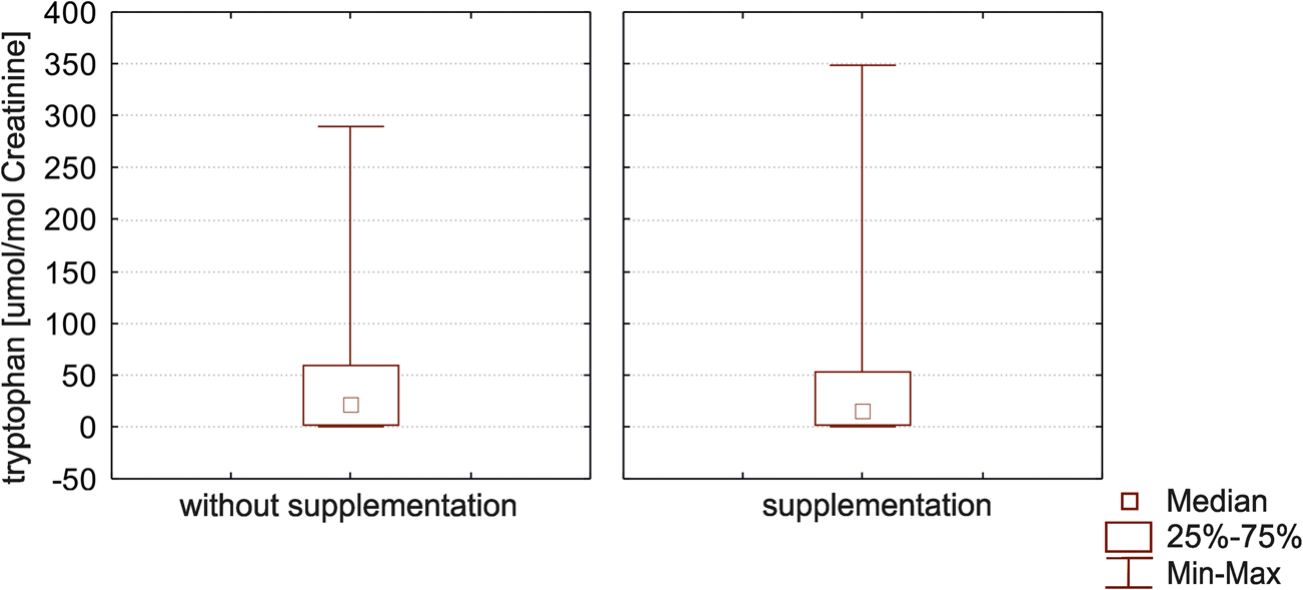
Box and Whisker plots for tryptophan level in both groups. In these box blots medians (not means) inside the 25–75% IC are represented. As better explained in the text, individuals without vitamin B and magnesium supplementation showed a higher variability inside the studied cohort

Table 4 shows the values found for urinary tryptophan in the entire study population of ASD group with vitamins B and magnesium supplementation and without the further supplementation. Two hundred thirty-six (236) subjects were recruited in the study. The level of urinary tryptophan in the group with the supplementation varied from 0.07 to 19.67 μmol/mmol creatinine, while in the second group from 0.01 to 348.94 μmol/mmol creatinine. The group with B vitamins and magnesium supplementation showed more stabilized values (Fig. 3, left panel) than the nutritionally supplemented group (Fig. 3, right panel), falling within the normal ranges. Values obtained for the group without supplementation were characterized by higher variability. This may suggest that supplementation would stabilize and ameliorate tryptophan metabolic balance, causing less excretion variability among subjects. Most of these values exceeded the normal ranges calculated in our labs for tryptophan excretion in urine. Figures 2 and 3 present the distribution of obtained results in the study population.

**Table 4:** Values obtained for quantification of tryptophan (μmol/mmol of creatinine) in urine samples of all the tested population of ASD children in group either with or without vitamins B and magnesium supplementation

	Mean	Standard deviation	Max	Min	Q25	Median	Q75	IC95
Supplementation	11.86	11.43	59.08	0.07	0.34	11.08	19.67	11.86 ± 5.14
Without supplementation	45.28	59.28	348.94	0.01	1.03	21.13	61.31	45.28 ± 7.89

Table 5 shows results obtained for urinary tryptophan in groups of ASD patients with different severity of symptoms with either supplementation of B vitamins and magnesium or without supplementation. The level of tryptophan varies considerably in patients with autistic disorder and Asperger syndrome. Additionally, the influence of the supplementation can be observed.

**Table 5:** Values obtained for quantification of tryptophan (μmol/mmol of creatinine) in urine samples of ASD children with diverse symptoms severity in group either with or without vitamins B and magnesium supplementation

ASD patients	Supplementation	Mean	Standard devotion	Q25	Median	Q75	IC95
Autistic disorder	yes	125.26	247.02	0.38	19.42	40.21	125.26 ± 130.74
	no	66.68	119.34	6.68	28.21	71.73	66.68 ± 17.81
Asperger syndrome	yes	1.71	3.08	0.27	0.36	0.46	1.71 ± 3.83
	no	19.44	34.55	0.24	0.52	29.16	19.44 ± 23.21
Autistic disorder and psychomotor retardation	yes	37.62	30.35	16.17	37.62	59.08	37.62 ± 272.65
	no	38.18	57.42	2.94	15.18	44.54	38.18 ± 36.48

The basic parameters of urine were within the IC_95_ parameters of normality in all ASD patients.

**Fig. 4:**
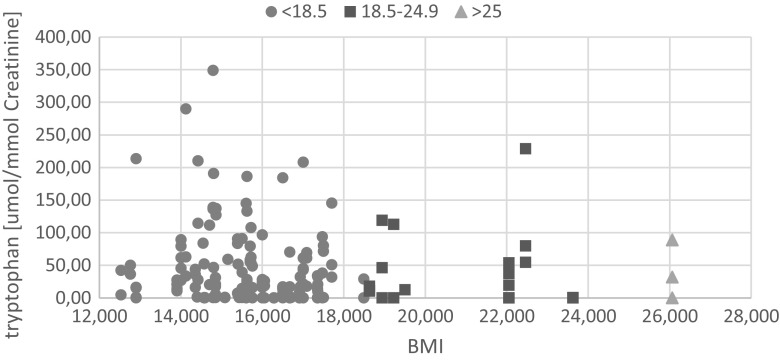
The dependence of the level of tryptophan from body mass index (BMI) value. The graph shows that, aside from some subject with values higher than 200 μmol/mmol Creat, highest BMI values are not associated with values ≥100 μmol/mmol Creat, suggesting that most probably BMI is not a good comorbid marker for Trp excretion impairment in ASD subjects

Application of the Shapiro-Wilk test showed that the hypothesis that data were normally distributed could be rejected (*p* < 0.05). Individual differences in the levels of tryptophan between the two groups were found after performing a Wilcoxon U-Mann–Whitney test. Considering the difference in the *p*-value of <0.05 to be statistically significant, the supplementation with vitamins B and magnesium turned out to have a great impact on the tryptophan levels. Additionally, it was found no correlation between the BMI value and the tryptophan concentration (Fig. 4).

## Discussion

Tryptophan is a precursor for biosynthesis of other crucial compounds. Thus, it can be speculated that abnormal level of this amino acid results in the incorrect formation of direct and indirect products of its metabolism, including serotonin and melatonin (Mann et al. 2001; Essa et al. 2013).

The present data showed that tryptophan levels in urine differ in groups with supplementation of B vitamins and magnesium and without supplementation. The vast majority of the results here reported is within usually previously reported reference range (Wishart et al. 2013) for ASD children with vitamins B and magnesium supplementation. Patients without such supplementation have disturbed levels of tryptophan. Moreover, the results obtained for children with autistic disorder and Asperger syndrome vary significantly. The cause of this may arise from different degree of autism severity. According to the current literature, patients with ASD are characterized by the reduced level of tryptophan (Boccuto et al. 2013; Naushad et al. 2013; Adams et al. 2011b). It is believed that it may arise from altered mitochondrial function (Boccuto et al. 2013; Rossignol and Frye 2012), and result in worsening of autistic symptoms, such as depression and irritability (Essa et al. 2013).

In the scientific literature, there are reports relating a reduced level of tryptophan in the ASD patients to irregularities of the metabolic pathways of this compound in the cells. Study of gene expression profiling in this field has indicated impaired functioning of the two genes: SLC7A5 and SLC7A8, which participates in the coding of tryptophan transporters. Furthermore, in individuals with ASD decreased expression of TPH2 gene was observed (Boccuto et al. 2013). This gene is responsible for encoding the tryptophan hydroxylase, which regulates the biosynthesis of serotonin (Boccuto et al. 2013). Inappropriate serotonin levels are correlated with psychological disorders, including depression, anxiety, obsessive-compulsive disorder, eating disorders, and even addiction (Mann et al. 2001; Essa et al. 2013). It should be noticed, that a vast majority of these disorders appear in ASD.

Many children with ASD also show sensitivity to foods, due to irregularities in the digestive system and/or the immune system. Incomplete digestion of food sugars, amino acids, fatty acids, etc., can cause the reaction of the immune system. The probability of such a reaction is increased in the case of simultaneous occurrence of inflammation in the body (Jyonouchi 2009; Adams 2013).

Patients with ASD often show abnormal levels of amino acids, and thus disturbed processes in which these compounds are involved. The cause of these abnormalities is food restrictions, restrictive diets low in protein, and impaired digestion (Adams 2013). Literature reports about abnormal levels of tryptophan (Arnold et al. 2003; Kałużna-Czaplińska et al. 2010), besides altered levels and expression of serotonin (Rolf et al. 1993), glutamic acid (Rolf et al. 1993), gamma-aminobutyric acid (GABA) (Rolf et al. 1993) and homocysteine (Kałużna-Czaplińska et al. 2011a; Kałużna-Czaplińska et al. 2013).

Furthermore, serotonin plays a great role in the formation of melatonin. The abnormal secretion of melatonin in people with ASD is described in a high number of scientific reports (Tordjman et al. 2005; Melke et al. 2008; Adams 2013), yet the cause of this disturbance and its relationship with ASD are still unknown. Literature reports that supplementation with this compound has brought improvement in autistic symptoms associated with sleep disorders in 66% of the study population. Only 8% were found to tighten these symptoms (Adams 2013).

Scientific interest is also focused on research based on the explanation of the effect of vitamin B-group supplementation (B6 and B12) on mitigation of ASD symptoms. Vitamin B6 is involved in nearly 1113 enzymatic reactions, including the production of neurotransmitters (serotonin and dopamine), glutathione (participation in detoxification) and hemoglobin (oxygen transport) (Adams 2013). Statistical analysis showed that the application of vitamins B2 and B6 in combination with magnesium results in lower levels of dicarboxylic acids including succinic acid, adipic acid, and octanedioic (or suberic) acid (Kałużna-Czaplińska et al. 2011b). It also postulated that the simultaneous taking vitamin B6 and magnesium results in an improved behavior of ASD patients (Rimland 1988; Martineau et al. 1985; Mousain-Bosc et al. 2006).

The present study revealed both increased and decreased level of tryptophan in ASD children without vitamins B and magnesium supplementation. This may result in the severity pattern reported for ASD symptoms. According to the current literature, abnormal levels of tryptophan entails further irregularities, including serotonin pathway. Compounds participating in it play a pivotal role in proper functioning of the body being responsible for basic mental and physiological activities. Additionally, the introduction of the supplementation leading to the normalization of tryptophan concentration was noted. No correlation between BMI and the level of this compound was found. Further investigation focused on the association between tryptophan level, and severity of autistic symptoms should be performed.

## Abbreviations

ATP: -5`(pyro)-triphosphates of adenosine
ASD: autism spectrum disorder
BIL: bilirubin
BMI: body mass index
CNS: central nervous system
BLD: erythrocytes
GABA: gamma-aminobutyric acid
GLU: glucose
IS: internal standard
KET: ketone
LEU: leukocytes
NAD: nicotinamide adenine dinucleotide
NIT: nitrite
PRO: protein
SG: specific gravity
TIC: total ion current
UBG: urobilinogen
